# Prevalence and Risk Factors for Rapid Eye Movement-Related Obstructive Sleep Apnea in Children

**DOI:** 10.3389/fped.2022.869986

**Published:** 2022-04-28

**Authors:** Surisa Chamnanpet, Prakarn Tovichien, Archwin Tanphaichitr, Wattanachai Chotinaiwattarakul

**Affiliations:** ^1^Department of Pediatrics, Faculty of Medicine Siriraj Hospital, Mahidol University, Bangkok, Thailand; ^2^Division of Pulmonology, Department of Pediatrics, Faculty of Medicine Siriraj Hospital, Mahidol University, Bangkok, Thailand; ^3^Department of Otorhinolaryngology, Faculty of Medicine Siriraj Hospital, Mahidol University, Bangkok, Thailand; ^4^Department of Medicine, Faculty of Medicine Siriraj Hospital, Mahidol University, Bangkok, Thailand; ^5^Siriraj Sleep Center, Faculty of Medicine Siriraj Hospital, Mahidol University, Bangkok, Thailand

**Keywords:** prevalence, riskfactor, REM sleep disorder, OSA, children

## Abstract

**Objectives:**

Different pathophysiological mechanisms and the distribution of respiratory events among rapid eye movement (REM) and non-rapid eye movement (NREM) sleep modulate the effect of obstructive sleep apnea (OSA). We aimed to study the prevalence and risk factors for REM-related OSA in children.

**Study Design:**

Retrospective, cross-sectional study.

**Methods:**

We recruited 366 children with OSA confirmed by polysomnography (PSG) over a 5-year period. REM-related OSA is defined by an obstructive apnea-hypopnea index (OAHI) in the REM sleep ≥2× than during NREM sleep.

**Results:**

The prevalence of REM-related OSA in children was 50.3%. Children with REM-related OSA were more likely to be female (*P* = 0.042), and had lower prevalence of adenotonsillar hypertrophy (*P* = 0.043) compared with children with other OSA subtypes. Children with REM-related OSA slept longer in the supine position (*P* = 0.003), had shorter duration of NREM1 sleep (*P* = 0.018), lower nadir SpO_2_ (*P* = 0.005), and a higher oxygen desaturation index 3% (ODI3%) (*P* = 0.014), and lower arousal index (*P* = 0.034) compared with other OSA subtypes. Female gender and supine sleep was the independent risk factors for REM-related OSA.

**Conclusion:**

The prevalence of REM-related OSA was 50.3%. OAHI_REM_ should be considered as an important parameter in future clinical research studies done in children with OSA.

## Introduction

Snoring is common in children. Obstructive sleep apnea (OSA) in children usually presents with habitual snoring, defined by snoring more than three nights/week. OSA arises from recurrent episodes of airway collapse during sleep that disrupts sleep architecture, ventilation, and cardiovascular homeostasis which leads to many consequences (1). Adverse consequences of pediatric OSA include neurobehavioral, cardiovascular, and endocrine consequences, which adversely affect the quality of life (2–5). The prevalence of OSA in children may be increasing due to the increasing prevalence of obesity and improved survival of children with chronic diseases such as neuromuscular disorders (6). Furthermore, obese children also have reduced REM sleep; this affects energy balance regulation and predisposes them to additional weight gain (7).

In America, the prevalence of habitual snoring has been reported to be 2.4–17.2%, and the prevalence of obstructive sleep apnea (OSA) was 1.2–5.7% (8). In Thailand in 2005, the prevalence of habitual snoring was reported to be 6.9–8.5%, and the prevalence of OSA was 0.7–1.3% (9). In 2016, the prevalence of OSA in children who underwent diagnostic polysomnography (PSG) at Chulalongkorn Memorial Hospital in Thailand was 92.7% and 40.4% of these children had severe OSA (10).

Human sleep is divided into four stages defined by electroencephalographic criteria. The main sleep stages include rapid eye movement (REM) sleep and non-REM (NREM) sleep (11). In children, OSA occurs predominantly in REM sleep (12, 13) possibly due to the reduced muscle tone, blunted arousal and ventilatory responses during this sleep stage (14, 15). Previous studies reported the prevalence of REM-predominant OSA ranging from 69.6 to 74.7%, while NREM-predominant OSA accounts for 16.7–25.3% of childhood and adolescent OSA (12, 13, 16, 17).

According to polysomnographic phenotypes, REM-related OSA is defined as a ratio of >2 OAHI in REM sleep (OAHI_REM_) compared with OAHI in non-REM (NREM) sleep (OAHI_NREM_) (18–20). The difference of pathophysiologic factors would predispose to airway obstruction in different sleep stages. These factors include upper airway collapsibility and muscle responsiveness, ventilatory control stability, and arousal threshold. All these factors change with age and disease status and may influence the pattern of polysomnographic phenotypes across age groups (21, 22). Sleep stage affects OSA severity and is influenced by many factors such as comorbidities including allergic rhinitis, asthma, and hypertension (23–27) but the natural history and clinical significance of sleep stage-dependent OSA have not been fully assessed. Because REM sleep plays a role in memory formation, processing of emotional information, neuronal plasticity and excitability (28). Therefore, respiratory disturbances in this sleep stages will have varying consequences, which are not rely only on an overall OAHI. Hence, we aimed to study the prevalence and risk factors for REM-related OSA in children.

## Materials and Methods

This retrospective cross-sectional study included children less than 18 years of age who underwent diagnostic polysomnography (PSG) for suspected OSA at the Siriraj Sleep Center between 2016 and 2020. OSA was diagnosed using clinical symptoms such as snoring, labored, paradoxical or obstructed breathing during sleep, sleepiness, hyperactivity, behavioral or learning problems, and an apnea-hypopnea index (AHI) from diagnostic polysomnography equal or more than 1 episode/h (29). We excluded children who had REM sleep less than 30 min during the diagnostic PSG. Before commencement of this study, the research protocol was approved by the Siriraj Institutional Review Board (approval no. Si 246/2563).

We collected demographic data from electronic medical records and PSG parameters from the official report. Weight was defined according to percentile weight for height-based and body mass index (BMI) z-score by age and gender. Overweight was defined as percentile weight for height greater than 120, or BMI z-score greater than or equal to 1. Obesity was defined as percentile weight for height greater than 140 or BMI z-score greater than or equal to two. Comorbidities and consequences of OSA were collected including adenotonsillar hypertrophy, allergic rhinitis, asthma, Down syndrome, neuromuscular disease, systemic hypertension, pulmonary hypertension, learning disorder, and attention deficit hyperactivity disorder (ADHD).

All PSG were scored by sleep specialists who were blinded to the condition of the child. Based on the PSG results, children were divided into three groups according to the polysomnographic phenotypes (23). REM-related and NREM-related OSA were defined as a ratio of OAHI in REM sleep (OAHI_REM_) to OAHI in NREM sleep (OAHI_NREM_) of >2 and <0.5, respectively. Children who did not meet these criteria were classified as stage-independent OSA. The OSA severity was classified based on OAHI: (1) mild OSA (AHI 1 to ≤5 events per hour), (2) moderate OSA (AHI > 5 to ≤10 events per hour), and (3) severe OSA (AHI > 10 events per hour). In addition, positional OSA was defined by OAHI in the supine position ≥2 × OAHI in the non-supine position (30).

Normally distributed data were expressed as mean and standard deviation, non-normally distributed data were expressed as median and interquartile range (25th, 75th percentile). Categorical data were expressed as frequency and percentage. Groups were compared using the unpaired t-test or Mann–Whitney U test, while categorical data were compared using the chi-squared test. Data were analyzed using IBM SPSS Statistics for Windows (version 22.0; IBM Corp., Armonk, NY, United States). A *P* value of <0.05 was considered statistically significant.

Binary logistic regression was performed to identify independent risk factors associated with REM-related OSA. The possible confounders with *P* < 0.2 in crude odd ratio (gender, adenotonsillar hypertrophy, asthma, supine total sleep time, NREM 1 sleep), allergic rhinitis, and OSA severity (OAHI) were analyzed to estimate an adjusted odd ratio.

## Results

Three hundred and sixty-six children were included in the analysis. One hundred and eighty-four (50.3%) children had REM-related OSA, 150 (41.0%) children had sleep stage independent OSA, and 32 (8.7%) children had NREM-related OSA. The median (interquartile range: IQR) age of the children was 8.0 (5.0, 12.0) years, 245 (66.9%) were male,%weight for height 121.00% (100.0%, 161.5%), and the BMI z-score was 1.4 (±2.5). Fifty-six children (15.3%) were overweight and 131 children (35.8%) were obese. Most children also had comorbidities. Common comorbidities included adenotonsillar hypertrophy 210 (57.4%), allergic rhinitis 130 (35.5%), asthma 39 (10.7%), Down syndrome 25 (6.8%), and neuromuscular disease 41 (11.2%). There was significant difference only in the prevalence of adenotonsillar hypertrophy by OSA subtype. Many children also had OSA consequences. Common consequences included attention deficit hyperactivity disorder (ADHD) 41 (11.2%), pulmonary hypertension 23 (6.3%), learning disorder 23 (6.3%), and systemic hypertension 18 (4.9%). There was a lower trend of children with REM-related OSA had systemic hypertension, but more of these children had pulmonary hypertension and learning disorders. Hence, there was no significant difference in the prevalence of consequences among each OSA subtypes (P = 0.864, 0.257 and 0.545, respectively) (Table 1).

**TABLE 1:** Demographic data of children with different OSA subtypes.

Data	Total *n* = 366	OSA subtypes			*P*-value
		REM-related *n* = 184	NREM-related *n* = 32	Sleep stage independent *n* = 150	
Age (year), median (IQR)	8.0 (5.0, 12.0)	8.0 (5.0, 12.0)	9.5 (7.3, 12.8)	8.0 (5.8, 12.0)	0.086
Female, n (%)	121 (33.1%)	70 (38.0%)	10 (31.3%)	41 (27.3%)	0.114
% Weight/Height, median (IQR)	121.0 (100.0, 161.5)	121.0 (100.0,164.0)	119.5 (97.3, 145.5)	123.5 (100.0,165.5)	0.589
BMI z-score, mean (SD)	1.4 (2.5)	1.3 (2.7)	1.1 (2.4)	1.4 (2.4)	0.864
**Comorbidities, n (%)**					
Adenotonsillar hypertrophy	210 (57.4%)	96 (52.2%)	13 (40.6%)	101 (67.3%)	0.003*
Allergic rhinitis	130 (35.5%)	69 (37.5%)	14 (43.8%)	47 (31.3%)	0.300
Asthma	39 (10.7%)	25 (13.6%)	4 (12.5%)	10 (6.7%)	0.118
Down syndrome	25 (6.8%)	15 (8.2%)	0 (0.0%)	10 (6.7%)	0.240
Neuromuscular diseases	41 (11.2%)	23 (12.5%)	4 (12.5%)	14 (9.3%)	0.640
**Consequences, n (%)**					
Systemic hypertension	18 (4.9%)	8 (4.4%)	2 (6.3%)	8 (5.3%)	0.864
Pulmonary hypertension	23 (6.3%)	14 (7.6%)	0 (0.0%)	9 (6.0%)	0.257
Learning disorder	23 (6.3%)	14 (7.6%)	2 (6.3%)	7 (4.7%)	0.545
ADHD	41 (11.2%)	22 (12.0%)	6 (18.8%)	13 (8.7%)	0.234

OSA, obstructive sleep apnea; REM, rapid eye movement; NREM, non-rapid eye movement; IQR, interquartile range; BMI, body mass index; ADHD, attention deficit hyperactivity disorder. *A p-value < 0.05 indicates statistical significance.

Ninety-two (25.1%) children had mild OSA, 121 (33.1%) had moderate OSA, and 153 (41.8%) had severe OSA. The OSA severity and proportion of children with positional OSA was not significantly different between REM-related OSA and other OSA subtypes (Table 2).

**TABLE 2:** Severity of REM related OSA in children comparing other phenotypes.

Severity	Total *n* = 366	REM-related *n* = 184	Others *n* = 182	*P*-value
Mild, *n* (%)	92 (25.1%)	44 (23.9%)	48 (26.4%)	0.846
Moderate, *n* (%)	121 (33.1%)	61 (33.2%)	60 (33.0%)	
Severe, *n* (%)	153 (41.8%)	79 (42.9%)	74 (40.7%)	
Positional OSA, *n* (%)	155 (42.3%)	80 (43.5%)	75 (41.2%)	0.660

*REM, rapid eye movement; OSA, obstructive sleep apnea.*

Children with REM-related OSA were more likely to be female (P = 0.042) and had lower prevalence of adenotonsillar hypertrophy (*P* = 0.043) compared with children with other subtypes. The other comorbidities and consequences were not significantly different in children with REM-related OSA compared with other subtypes (Table 3).

**TABLE 3:** Demographic data of children with REM-related OSA and other subtypes.

Data	Total *n* = 366	REM *n* = 184	Others *n* = 182	*P*-value
Age (year), median (IQR)	8.0 (5.0, 12.0)	8.0 (5.0, 12.0)	9.0 (6.0, 12.0)	0.168
Female, *n* (%)	121 (33.1%)	70 (38.0%)	51 (28.0%)	0.042*
% Weight/Height, median (IQR)	121.0 (100.0, 161.5)	121.0 (100.0, 164.0)	122.0 (100.0, 161.5)	0.755
BMI z-score, mean (SD)	1.4 (2.5)	1.4 (2.7)	1.4 (2.4)	0.979
**Comorbidities, n (%)**				
Adenotonsillar hypertrophy	210 (57.4%)	96 (52.2%)	114 (62.6%)	0.043*
Allergic rhinitis	130 (35.5%)	69 (37.5%)	61 (33.5%)	0.426
Asthma	39 (10.7%)	25 (13.6%)	14 (7.7%)	0.068
Down syndrome	25 (6.8%)	15 (8.2%)	10 (5.5%)	0.314
Neuromuscular diseases	41 (11.2%)	23 (12.5%)	18 (9.9%)	0.429
**Consequences, n (%)**				
Systemic hypertension	18 (4.9%)	8 (4.4%)	10 (5.5%)	0.620
Pulmonary hypertension	23 (6.3%)	14 (7.6%)	9 (4.9%)	0.294
Learning disorder	23 (6.3%)	14 (7.6%)	9 (4.9%)	0.294
ADHD	41 (11.2%)	22 (12.0%)	19 (10.4%)	0.645

REM, rapid eye movement; IQR, interquartile range; BMI, body mass index; ADHD, attention deficit hyperactivity disorder. *A p-value < 0.05 indicates statistical significance.

Children with REM-related OSA slept longer in the supine position (*P* = 0.003), had shorter duration of NREM1 sleep (*P* = 0.018), lower nadir SpO_2_ (*P* = 0.005) and higher oxygen desaturation index 3% (ODI3%) (*P* = 0.014), and lower arousal index (*P* = 0.034) compared with children with other subtypes (Table 4).

**TABLE 4:** Comparison of polysomnographic parameters between REM-related OSA versus other subtypes.

PSG parameters, median (IQR)	Total *n* = 366	REM-related *n* = 184	Other subtypes *n* = 182	*P*-value
Sleep efficiency (%)	92.4 (87.3, 95.4)	92.4 (88.0, 95.5)	92.5 (87.1, 95.4)	0.724
Sleep onset latency (min)	9.0 (4.0, 19.0)	10.0 (3.0, 21.0)	9.0 (4.0, 16.0)	0.513
REM onset latency (min)	157.0 (110.0, 200.0)	155.0 (103.0, 206.0)	158.0 (115.0, 193.0)	0.737
Wake after sleep onset (min)	21.0 (11.0, 44.0)	19.0 (10.0, 38.0)	22.0 (12.0, 49.0)	0.113
Total sleep time (min)	417.0 (397.0, 442.0)	417.0 (397.0, 436.0)	417.0 (398.0, 447.0)	0.445
Supine total sleep time (min)	284.0 (199.0, 366.0)	298.0 (204.0, 379.0)	259.0 (177.0, 344.0)	0.003*
Side total sleep time (min)	135.0 (47.0, 231.0)	111.0 (25.0, 210.0)	162.0 (70.0, 247.0)	0.001*
NREM 1 (%)	4.0 (2.0, 7.2)	3.4 (1.8, 6.0)	4.8 (2.0, 8.5)	0.018*
NREM 2 (%)	46.4 (37.4, 54.0)	46.4 (37.0, 54.7)	46.9 (37.9, 53.5)	0.821
NREM 3 (%)	29.7 (22.3, 39.1)	30.5 (23.4, 38.9)	28.7 (21.6, 39.3)	0.332
REM (%)	16.4 (13.4, 20.0)	16.8 (13.8, 20.5)	16.1 (13.3, 19.3)	0.103
OAHI (events per hour)	8.4 (4.9, 15.2)	8.8 (5.0, 15.1)	8.2 (4.6, 15.5)	0.656
Nadir SpO2 (%)	88.0 (83.0, 91.0)	88.0 (81.0, 91.0)	89.0 (84.0, 92.0)	0.005*
ODI3% (events per hour)	4.9 (1.7, 9.6)	6.1 (2.4, 10.5)	3.4 (1.5, 9.3)	0.014*
Average ETCO2 (mmHg)	43.0 (39.0, 45.0)	43.0 (40.0, 46.0)	42.0 (39.0, 45.0)	0.134
Arousal index (events per hour)	9.9 (7.3, 15.1)	9.6 (7.1, 13.3)	11 (7.5, 16.3)	0.034*

REM related OSA, rapid eye movement related obstructive sleep apnea; OSA, obstructive sleep apnea; REM, rapid eye movement; IQR, interquartile range; NREM, non-rapid eye movement; OAHI, obstructive apnea-hypopnea index; SpO2, oxygen saturation; ODI3%, oxygen desaturation index 3%; ETCO2, End tidal carbon dioxide. *A p-value < 0.05 indicates statistical significance.

After adjusting for covariables, we found female gender was a significant risk factor for REM-related OSA with an adjusted odds ratio of 1.874 (95% CI, 1.171–2.999) (*P* = 0.009). Duration of supine sleep was also another significant risk factor for REM-related OSA with an adjusted odds ratio of 1.003 (95% CI, 1.001–1.005) (*P* = 0.005) (Table 5).

**TABLE 5:** Odd ratio, 95% confidence interval and *p*-value of REM-related OSA and other subtypes for demographic data, comorbidity, consequence, and polysomnographic parameters.

Data	Crude OR (95% CI)	*p*-Value	Adjusted OR (95% CI)	*p*-Value
Age, years	0.971 (0.923, 1.022)	0.262		
Female	1.577 (1.016, 2.448)	0.042*	1.874 (1.171, 2.999)	0.009*
% Weight/Height	1.000 (0.995, 1.005)	0.886		
BMI z-score	0.999 (0.921, 1.083)	0.979		
**Comorbidities**				
Adenotonsillar hypertrophy	0.651 (0.429, 0.987)	0.043*	0.692 (0.442, 1.084)	0.108
Allergic rhinitis	1.190 (0.775, 1.827)	0.426	1.035 (0.655, 1.637)	0.881
Asthma	1.887 (0.947, 3.759)	0.071	1.839 (0.882, 3.833)	0.104
Down syndrome	1.527 (0.667, 3.493)	0.317		
Neuromuscular diseases	1.302 (0.677, 2.503)	0.430		
**PSG parameters**				
Supine total sleep time	1.003 (1.001, 1.004)	0.006*	1.003 (1.001, 1.005)	0.005*
NREM 1,%	0.927 (0.884, 0.972)	0.002*	0.923 (0.877, 0.970)	0.002*
OAHI	0.991 (0.974, 1.009)	0.350	1.004 (0.984, 1.025)	0.668

REM-related OSA, rapid eye movement related obstructive sleep apnea; BMI, body mass index; NREM, non-rapid eye movement, OAHI, obstructive apnea-hypopnea index. *A p-value < 0.05 indicates statistical significance.

## Discussion

The prevalence of REM-related OSA in our study was 50.3%, lower than previous study which reported at 65% (17). In the past, there was significant heterogeneity in defining REM-predominant OSA. Previous studies have reported 69% of OSA children had higher OAHI_REM_ than OAHI_NREM_ (12, 13), and 55% of obstructive apneas in children occurred during REM sleep (31). Goh et al. reported that the apnea index, apnea duration and degree of desaturation in REM sleep were greater than during NREM sleep (31). Withdrawal of excitatory noradrenergic and serotonergic inputs to pharyngeal motor neurons during REM sleep predisposes to upper airway collapse (32). OSA is particularly problematic in REM sleep in children due to muscle atonia, reduced ventilatory response to hypoxia and hypercapnia, and reduced arousal threshold (33).

In adults, the overall AHI is more influenced by the NREM AHI because of longer duration of NREM sleep. Moreover, as the severity of OSA increases, the duration of REM sleep reduces (34). In adults with OSA, the prevalence of REM-related OSA is 10–36% (18–20) and respiratory events with more severe clinical phenotypes occur predominantly during NREM sleep (35–37). Severe sleep fragmentation is characteristic of this subtype (38) in contrast to REM-predominant type in children where sleep architecture is reported to be preserved (31). This pattern was also reported in up to a third of children with moderate to severe OSA, especially in older children with high arousal indices and less severe desaturations with events (13). Thus, different pathophysiological mechanisms will be operational in these two OSA subtypes. Hormonal changes during puberty or thereafter might lead to the reversal of REMS-NREMS OSA predominance. However, recent study revealed that the majority (72%) of children with REM-related OSA at baseline and persistent OSA at 10-year follow-up remained to have REM-related OSA (17). Further study of the longitudinal changes during the transition to adulthood may shed light on this issue, particularly considering the higher risk of OSA recurrence after adolescence in children who were previously diagnosed and treated for OSA during childhood (39).

We observed that OSA severity was not significantly different by OSA subtype nor was the proportion of children with positional OSA. Positional (supine dependent) OSA is less common in children compared to adults. It is defined by an OAHI in the supine position ≥2 × OAHI in the non-supine position. Previous study reported the prevalence of positional OSA in children at 18.7%. They also found children with positional OSA were significantly older, had a high prevalence of obesity, and a lower tonsil score (30). We found the prevalence of positional OSA at 42.3%, much higher than previous study. It may be affected by the high prevalence of obese children in our study. Although OSA worsens during REM sleep, this was not observed for positional OSA. Furthermore, our study also found no difference in the proportion of positional OSA between children with REM-related OSA and others.

Individual variations in endotypes that predispose to airway obstruction in different sleep stages may affect polysomnographic phenotypes. A recent study revealed that individuals with REM-related OSA showed a significantly more collapsible airway in REM compared with NREM sleep, while individuals with NREM-related OSA had a higher loop gain and lower ventilatory drive during obstructed breathing at arousal threshold during NREM sleep (21).

The effect of sleep stage on OSA severity might be influenced by many factors such as body weight and by comorbidities including obesity, allergic rhinitis and asthma (24–26). Obese children have reduced REM sleep; this affects energy balance regulation and predisposes them to additional weight gain (7). REM sleep also influences upper and lower airway disorders in children. Children with rhinitis and OSA tend to have more REM-related upper airway obstruction (OAHI_REM_), and those with asthma and OSA seem to have more REM-related hypoxemia (25).

Our study showed that the prevalence of asthma in children with REM related-OSA tended to be higher than other subtypes, but did not reach statistical significance. Gutierrez et al. reported that asthma is associated with REM-related breathing abnormalities in children with moderate to severe OSA, and the link between asthma and REM-related OSA is independent of asthma control and obesity (24). OSA is also associated with nasal inflammatory changes (40). Although REM sleep is characterized by significant nasal congestion (41, 42) and children with rhinitis have more REM-related upper airway obstruction independent of confounding factors (25), we did not observe a meaningful difference in the proportion of children with allergic rhinitis between REM-related OSA and other subtypes. In contrast, Huseni et al. found that children with rhinitis have more REM-related OSA independent of age, BMI, gender, ethnicity and atopic status (25). Furthermore, we found that the prevalence of children with adenotonsillar hypertrophy in REM-related OSA was significantly lower than with other subtypes.

The different functions of REM and NREM sleep might result in different clinical outcomes. The distribution of respiratory events among REM and NREM sleep may modulate the effect of OSA in children. As apnea tends to be longer and desaturation more severe in REM sleep, we hypothesize that the consequences of OSA may be different if the events occur predominantly in REM sleep. At birth, a term infant has equal proportions of REM and NREM sleep. REM sleep duration decreases with increasing age. The proportion of REM sleep at 3–5 years of age is 25% that of adults (43). Therefore, children with obstructive events predominantly in REM sleep are more vulnerable to adverse neurocognitive outcomes for a given OAHI.

The clinical significance of REM-related OSA is controversial. Most adult studies demonstrate that REM-predominant OSA is associated with excessive daytime sleepiness, non-adherence to continuous positive airway pressure, depression, hypertension, non-dipping of nocturnal blood pressure, increased insulin resistance, and impaired spatial navigational memory (44–51). However, results are not consistent across all studies. Some studies also suggest that OAHI_NREM_ is more often associated with adverse consequences than OAHI_REM_ (37, 52–54). REM sleep is important for memory consolidation and learning (55, 56). Therefore, a higher proportion of respiratory events in REM sleep may be linked to adverse neurocognitive outcomes of OSA.

Pediatric OSA is associated with elevated blood pressure (57–60); thus, the association between blood pressure and REM-related OSA in children is controversial (23, 27). Moderate to severe OSA has a significant effect on pulse transit time during REM sleep, suggesting that these children may have a higher baseline blood pressure during this state (27). In contrast, Au et al. found children with REM-related OSA had a significantly lower daytime and nighttime systolic blood pressure (SBP) than children with stage independent OSA (23). Moreover, OAHI_NREM_, but not OAHI_REM_, was significantly associated with both daytime and nighttime SBP after controlling for age, gender, and body size. They postulated children with obstructive events mainly in REM sleep may have less cardiovascular complications than those with stage-independent OSA (23). However, recent study revealed that REM-related OSA was associated with higher nocturnal BP and a lesser degree of nocturnal SBP dipping in children (17). In our study, we did not find significant differences in the proportions of children with systemic hypertension between OSA subgroups. REM sleep is associated with greater sympathetic activity and cardiovascular instability than NREM sleep in both healthy subjects and patients with OSA (61). Therefore, respiratory events during REM sleep in REM-related OSA could play an important role in conferring a higher cardiovascular risk (44).

We observed that children with REM-related OSA had longer sleep in the supine position, shorter sleep duration in NREM1, and lower SpO_2_ nadir and higher ODI3% than children with other OSA subtypes. The severity of intermittent hypoxia would increase sympathetic activity, endothelial dysfunction, and systemic inflammation (62).

While our study confirms that OSA is predominantly a REM sleep-related disorder in the majority of children, we identified a significant proportion of other OSA subtypes. This difference of pathophysiology and outcomes has implications for diagnostic monitoring. Logistic regression analysis revealed that female gender and supine sleep was a significant predictor of REM-related OSA after adjustment for other confounders.

The strengths of our study are the relatively large sample size and the use of objective data to evaluate OSA variables. Our findings highlight the importance of sleep stage distribution of respiratory events in studies of the pathophysiology and outcomes of OSA in children. All PSG were scored by sleep specialists who were blinded to the condition of the child. However, the cross-sectional design of our study prevents us from arriving at any type of cause-effect conclusion. Follow-up is needed to know the treatment effect on REM-related OSA and whether it is similar to the NREM-related OSA subgroup or not. Therefore, we recommended further study in specific population at risk of REM-related OSA to confirm our hypothesis. New insights may allow for the discovery of mechanistic pathways that connect with sleep biology, which in turn, may result in novel strategies for the treatment of pediatric OSA.

## Conclusion

The prevalence of REM-related OSA in children from our study was 50.3%. Female gender and supine sleep were independent risk factors for REM-related OSA in children. OAHI_REM_ should be considered as an important parameter in future clinical research studies done in children with OSA.

## Data Availability Statement

The raw data supporting the conclusions of this article will be made available by the authors, without undue reservation.

## Ethics Statement

The studies involving human participants were reviewed and approved by the Siriraj Institutional Review Board. Written informed consent from the participants’ legal guardian/next of kin was not required to participate in this study in accordance with the national legislation and the institutional requirements.

## Author Contributions

SC performed data collection, statistical analysis, and manuscript preparation. PT contributed to study conception, study design, statistical analysis, and manuscript preparation. AT and WC critically revised the manuscript for important intellectual content. All authors contributed to the article and approved the submitted version.

## Conflict of Interest

The authors declare that the research was conducted in the absence of any commercial or financial relationships that could be construed as a potential conflict of interest.

## Publisher’s Note

All claims expressed in this article are solely those of the authors and do not necessarily represent those of their affiliated organizations, or those of the publisher, the editors and the reviewers. Any product that may be evaluated in this article, or claim that may be made by its manufacturer, is not guaranteed or endorsed by the publisher.
